# Protein arginine methyltransferase 5: A novel therapeutic target for triple‐negative breast cancers

**DOI:** 10.1002/cam4.2114

**Published:** 2019-04-08

**Authors:** Mathilde Vinet, Samyuktha Suresh, Virginie Maire, Clarisse Monchecourt, Fariba Némati, Laetitia Lesage, Fabienne Pierre, Mengliang Ye, Auriane Lescure, Amélie Brisson, Didier Meseure, André Nicolas, Guillem Rigaill, Elisabetta Marangoni, Elaine Del Nery, Sergio Roman‐Roman, Thierry Dubois

**Affiliations:** ^1^ Translational Research Department Institut Curie, PSL Research University Paris France; ^2^ Breast Cancer Biology Group Institut Curie Paris France; ^3^ Preclinical Investigation Laboratory Institut Curie Paris France; ^4^ Platform of Investigative Pathology, Department of Pathology Institut Curie Paris France; ^5^ Biophenics High‐Content Screening Laboratory, Cell and Tissue Imaging Facility (PICT‐IBiSA) Institut Curie Paris France; ^6^ Institute of Plant Sciences Paris‐Saclay (IPS2), UMR 9213, UMR1403, CNRS, INRA Université Paris‐Sud, Université d'Evry, Université Paris‐Diderot, Sorbonne, Paris‐Cité Orsay France; ^7^ Laboratoire de Mathématiques et Modélisation d'Evry (LaMME) Université d'Evry Val d'Essonne, UMR CNRS 8071, ENSIIE, USC INRA Evry France

**Keywords:** breast cancer, molecular biology, targeted therapy, translational research

## Abstract

TNBC is a highly heterogeneous and aggressive breast cancer subtype associated with high relapse rates, and for which no targeted therapy yet exists. Protein arginine methyltransferase 5 (PRMT5), an enzyme which catalyzes the methylation of arginines on histone and non‐histone proteins, has recently emerged as a putative target for cancer therapy. Potent and specific PRMT5 inhibitors have been developed, but the therapeutic efficacy of PRMT5 targeting in TNBC has not yet been demonstrated. Here, we examine the expression of PRMT5 in a human breast cancer cohort obtained from the Institut Curie, and evaluate the therapeutic potential of pharmacological inhibition of PRMT5 in TNBC. We find that PRMT5 mRNA and protein are expressed at comparable levels in TNBC, luminal breast tumors, and healthy mammary tissues. However, immunohistochemistry analyses reveal that PRMT5 is differentially localized in TNBC compared to other breast cancer subtypes and to normal breast tissues. PRMT5 is heterogeneously expressed in TNBC and high PRMT5 expression correlates with poor prognosis within this breast cancer subtype. Using the small‐molecule inhibitor EPZ015666, we show that PRMT5 inhibition impairs cell proliferation in a subset of TNBC cell lines. PRMT5 inhibition triggers apoptosis, regulates cell cycle progression and decreases mammosphere formation. Furthermore, EPZ015666 administration to a patient‐derived xenograft model of TNBC significantly deters tumor progression. Finally, we reveal potentiation between EGFR and PRMT5 targeting, suggestive of a beneficial combination therapy. Our findings highlight a distinctive subcellular localization of PRMT5 in TNBC, and uphold PRMT5 targeting, alone or in combination, as a relevant treatment strategy for a subset of TNBC.

## 1 INTRODUCTION

The efficacy of breast cancer therapeutic management has considerably improved in recent years, however, the subgroup of patients with triple‐negative breast cancers (TNBC), defined by the absence of expression of estrogen (ER) and progesterone (PR) receptors and of HER2 overexpression, maintain a poor prognosis.^1^ One of the major problematics in TNBC therapeutic management is the heterogeneity of the disease, and the absence of clear molecular targets.^2^ To account for this heterogeneity, several groups have classified TNBC into distinct subtypes based on DNA, RNA, epigenetic and proteomic profiling, with the aim of providing therapeutic guidance. TNBC patients generally respond well to conventional chemotherapies, but suffer high recurrence rates due to residual, resistant tumor cells, and continually represent a large proportion of breast cancer deaths. TNBC thus remain a major challenge for oncologists, and the development of alternative treatments is warranted to bypass resistance to chemotherapies and improve patient survival rates.^3^, ^4^

Protein arginine methylation is a key post‐translational modification implicated in gene transcription and signal transduction.^5^ Protein arginine methyltransferase 5 (PRMT5) is the main type II PRMT, which catalyzes the symmetric dimethylation of arginine residues of histone and non‐histone proteins.^6^ PRMT5 functions as part of a complex coined the methylosome, along with its binding partner and co‐activator methylosome protein 50 (MEP50). PRMT5 is overexpressed in a number of cancers including melanoma, multiple myeloma, glioblastoma, lung, gastric, prostate, ovarian, and colorectal cancers,^6^ and high expression of PRMT5 often correlates with poor patient prognosis.^6^

Moreover, PRMT5 regulates the expression and activity of key players in oncogenic and apoptotic signaling, and was shown to participate in stem cell maintenance.^6^, ^7^ PRMT5‐mediated H3R8 and H4R3 methylation, for example, repress the transcription of a number of tumor suppressors including RB‐family genes, ST7, and NM23, leading to increased cell survival and proliferation.^8^, ^9^ PRMT5 also directly methylates p53, PI3K, and E2F‐1, thereby influencing the transcriptional activity of these essential cell fate regulators to promote cell growth and inhibit apoptosis.^10^, ^11^, ^12^ Given this, PRMT5 has been attributed oncogenic functions and has recently received considerable attention as a potential therapeutic target in cancer. Several selective and potent small‐molecule inhibitors have been developed against PRMT5 and their effects on cancer development are now being assessed in vitro, in vivo,^13^, ^14^, ^15^ as well as in a clinical trial.^15^, ^16^ Among these is the inhibitor EPZ015666, which competes with the PRMT5 peptide substrate binding pocket to impede PRMT5‐substrate interaction and subsequent methylation.^13^ In this study, we evaluate the therapeutic potential of PRMT5 inhibition in TNBC in vitro and in vivo using the specific and potent inhibitor EPZ015666,^13^ and analyze the expression and localization of PRMT5 in a cohort of human breast cancer biopsies.

## 2 MATERIALS AND METHODS

### 2.1 Human samples, transcriptome microarray, and immunohistochemistry

Our cohort has been described previously.^17^ Briefly, transcriptome microarray (U133 Plus 2.0 Affymetrix chips) was performed on TNBC (n = 41), HER2+/ER− (n = 30), luminal A (LA, n = 29), luminal B (LB, n = 30), and normal human samples (n = 11).^17^ Immunohistochemistry (IHC) was performed as described 17, 18 on the following number of tumors (TNBC: n = 41; HER2+/ER−: n = 29; LA: n = 22; LB: n = 27) and on normal breast tissues (n = 7). For PRMT5 staining, tissue microarrays (TMA) containing alcohol, formalin and acetic acid (AFA)‐fixed paraffin‐embedded tissues were made as described.^17^, ^18^ Antigen retrieval was performed in EDTA buffer pH = 6 (PRMT5). The PRMT5 antibody (Table S1) was validated for IHC using cell pellets fixed in the same way than the tumors from cell lines depleted or not of PRMT5. To assess whether the mean percentage of stained cells differs between any two subtypes, we performed Student *t* tests.

The TCGA breast invasive carcinoma (TCGA‐BRCA) cohort is publicly available.^19^ The RNA‐SeqV2 Level 3 data (Jan 2015) were downloaded from the TCGA Research Network (http://cancergenome.nih.gov/) and integrated into a platform in knowledge data integration (KDI) at Institut Curie (https://bioinfo-portal.curie.fr). Subtype classification was based on immunohistochemical status for the estrogen receptor (ER), progesterone receptor (PR) and HER2, as follows. TNBC: ER−, PR− and HER2‐negative (n = 157); HER2+/ ER−: ER− and PR‐negative, HER2‐positive (n = 41); luminal B: ER− and/or PR‐positive, HER2‐positive (n = 153); luminal A: ER− and/or PR‐positive, HER2‐negative (n = 663). The TCGA database includes 113 referenced normal breast tissue samples.

### 2.2 Cell culture

Cell lines were purchased between 2005 and 2009 from the American Type Culture Collection (ATCC, LGC Promochem) and authenticated by short tandem repeat profiling in 2018, using the Powerplex 16 system (Promega). All cell lines were cultured as described.^20^, ^21^ MDA‐MB‐468 cells were cultured in RPMI‐1640 (LifeTechnologies) supplemented with 10% (vol/vol) fetal bovine serum (FBS, LifeTechnologies), 100 U/mL penicillin and 100 µg/mL streptomycin (P/S, LifeTechnologies). HCC38, HCC70, HCC1937, and HCC1954 cells were cultured using the same media, complemented with 1.5 g/L sodium bicarbonate (LifeTechnologies), 10 mmol/L Hepes (LifeTechnologies), and 1 mmol/L sodium pyruvate (LifeTechnologies). MDA‐MB‐157 and Hs578‐T cells were cultured in DMEM (Life Technologies) supplemented with 10% FBS and 1%P/S. MCF‐10A and MCF‐12A cells were cultured in the same media, supplemented with 0.01 mg/mL insulin, 100 ng/mL cholera toxin (Sigma), 500 ng/mL hydrocortisone (SERB Laboratories), and 20 ng/mL epidermal growth factor (Sigma). MDA‐MB‐453 cells were cultured in DMEM‐F12 (LifeTechnologies) supplemented with 10% FBS and 1%P/S. BT‐20 and MCF‐7 cells were cultured in MEM (Sigma‐Aldrich) containing 10% FBS, 1% P/S, 1.5 g/L sodium bicarbonate, 0.1 mmol/L non‐essential amino‐acids (NEAA, LifeTechnologies) and 1 mmol/L sodium pyruvate. SK‐BR‐3 cells (HTB‐30) were cultured in McCoy5a (LifeTechnologies) containing 10% FBS and 1% P/S. All cell lines were maintained at 37°C in a humidified atmosphere with 5% CO2.

### 2.3 PRMT5 inhibitors, antibodies, and small interfering RNAs (siRNAs)

PRMT5 inhibitor EPZ015666 was purchased from Clinisciences and DC Chemicals. EPZ015938 was purchased from Selleckchem. Antibodies used are listed in Table S1. All siRNAs were purchased from Qiagen: Allstars negative control (SI03650318); PRMT5_1 (SI04216492), target sequence 5′‐TGCCGTGGTGACGCTAGAGAA‐3′; PRMT5_2 (SI04248951), target sequence 5′‐CAGAGATCCTATGATTGACAA‐3′; PRMT5_3 (SI04308416), target sequence 5′‐CTGGCGATGCAGCAATTCCAA‐3′; PRMT5_4 (SI00719432), target sequence 5′‐CAGCCCATAACGGTACGTGAA‐3′.

### 2.4 Cellular assays

Cell assays were performed as already described.^17^, ^18^, ^20^, ^21^, ^22^ Briefly, cells were incubated with DMSO or a PRMT5 inhibitor (EPZ015666, EPZ015938), or transfected with 40 nmol/L siRNA (Qiagen) using INTERFERin (Polyplus Transfection) (BT‐20, Hs578T, MCF‐10A, MDA‐MB‐453, MDA‐MB‐468) or Lipofectamine RNAiMAX (Life Technologies) (HCC38, HCC70). Cell proliferation determined by MTT (Sigma). Apoptotic activity was determined by the Caspase‐Glo 3/7 luminescent assay (Promega) or by Western blot analysis. Caspase activity using the luminescent assay was normalized to cell viability, measured by a concomitant MTT assay. Cell‐cycle analysis was carried out with LSRII (Becton Dickinson) using BD FACSDIVA SoftwareTM (BD Bioscience) to determine cellular DNA content, and analyzed using FlowJo and Modfit LT softwares. For the colony formation assay, cells were treated with drugs or siRNA, and incubated for 5 (MCF10A), 9 (MDA‐MB‐468) or 14 days (BT20, HCC38, HCC70, MDA‐MB‐453). Colonies were then stained with a solution containing 0.05% Coomassie Brilliant Blue R‐250, 50% methanol, 10% acetic acid, 40% ultrapure water for 20 minutes and rinsed with water. For the mammosphere formation assay, 2000 HCC38 cells were seeded in six‐well ultra‐low attachment plates (Corning, VWR, ref. 734‐1582) and cultured in MEBM basal medium (Lonza) supplemented with 1% B27 (Invitrogen), 4 µg/mL insulin, 2 µg/mL hydrocortisone (SERB Laboratories), 20 ng/mL epidermal growth factor (Sigma), 10 µmol/L 2‐mercaptoethanol (Invitrogen), 100 U/mL penicillin and 100 µg/ml streptomycin (P/S, LifeTechnologies). The number of mammospheres in each well was counted under a microscope after 14 days. All the experiments were repeated at least three times.

### 2.5 Screening of the Prestwick drug library

MDA‐MB‐453 cells were seeded into 384‐well plates (ViewPlate‐384 Black Perkin Elmer) in 40 µL of media, using a MultiDrop combi (Thermo Fisher Scientific). Twenty‐four hours later, cells were incubated with 1200 clinically licensed compounds from the Prestwick Chemical Library (Prestwick Chemical) (final concentration: 10µmol/L), or one of six additional compounds were added (Table S2; later referred to as “Prestwick” along with the Prestwick Chemical library drugs; final concentration as indicated), mixed or not with EPZ015666 (DC Chemicals) (final concentration: 10 µmol/L). Liquid handling was performed using the MultiChannel Arm™ 384 (MCA 384) (TECAN). For controls, DMSO alone (0.5%) and EPZ015666 (10 µmol/L) were added to the cells as single agents. Cell viability was monitored using the CellTiter‐Glo (Promega) assay after 96 hours using a CLARIOStar (BMG Labtech). The experiment was carried out in duplicates. Positive hits for each compound were identified as follows: data were first transformed with log functions; B‐score normalization was then applied to each replicate separately, and includes corrections for plates, rows, and columns. Median and median absolute deviation (MAD) were computed and used to calculate Robust Z‐scores (RZ‐scores) for each sample, according to the formula: score = (value − median)/(1.4826 × Median MAD). RZ‐scores were calculated for the comparison of each compound against the DMSO‐treated cells. A compound was identified as a ‘hit', if the RZ‐score was <−2 in the two replicates. The correspondence between RZ‐score and cell proliferation is given by the following formula:

$$\%proliferation=\frac{exp(RZscore\times 1,4826\times MAD+median\left(alltreatedwells\right))}{exp(median\left(alltreatedwells\right))}\times 100$$

A $\Delta RZscore=RZscore\left(Prestwick+EPZ015666\right)-RZscore\left(Prestwick\right)$ was calculated for each ‘hit' to quantify the effect of the Prestwick + EPZ015666 drug combination.

### 2.6 Combination analysis

Cells were seeded into 96‐well plates and treated with various concentrations ranging from 0 to 5 or 10 µmol/L of EPZ015666 (DC Chemicals) and/or Erlotinib (Cayman Chemical) or with DMSO alone after 24 hours. Cell viability was determined after 3 days (MDA‐MB‐453), as in the screen for experimental validation, or 7 days (BT‐20, HCC70, MDA‐MB‐468, HCC38) by CellTiter‐Glo (CTG, Promega) assay. Luminescent signals were measured using an Infinite 200 spectrophotometer (Tecan). Chalice Analyzer (http://chalice.horizondiscovery.com/analyzer-server/cwr/analyze.jsp) was used to calculate the Loewe excess. Synerdrug Analyzer (https://github.com/bioinfo-pf-curie/synerdrug) was used to calculate Chou‐Talalay Combination Indexes. Experiments were repeated at least three times.

### 2.7 Mice, compounds, treatment, and tumor growth measurement

Six‐week‐old Female Swiss nude mice were purchased from Charles River (Les Arbresles, France) and maintained in specific pathogen‐free conditions. Their care and housing were in accordance with institutional guidelines as put forth by the French Ethical Committee. EPZ015666 (DC Chemicals) was formulated at 1 mg/mL in 0.5% Methylcellulose (Sigma Aldrich) + 0.5% Tween 20 (Sigma Aldrich). EPZ015666 toxicity studies were performed by administration of 100 mg/kg, per‐os (po), twice daily, 5 days per week, to nude mice. Treatment was not associated with any mortality or body weight loss (Figure S1). The patient‐derived xenograft model HBCx‐17 was established from a triple‐negative breast cancer as detailed elsewhere^23^, ^24^ and chosen on the basis of high mRNA expression of PRMT5. Briefly, tumor fragments (30‐60 mm^3^) were grafted into the inter‐scapular fat pad of nude mice. When tumors reached 60‐100 mm^3^ (day 1 of the analysis), mice were randomly assigned to control or treatment groups (n = 7/group). Tumor volume was evaluated by measuring two perpendicular tumor diameters with a caliper, twice a week, as described.^17^, ^23^ Mice were ethically killed at the end of the experiment (5 weeks).

### 2.8 Statistical analyses

For caspase activity assay, sub‐G1 cell cycle analysis, colony formation, and mammosphere formation assays, differences between groups were assessed using Student *t* tests and were considered significant if the *P* value was below 0.05. For the cell cycle experiment, we used cell counts to evaluate the difference between DMSO‐treated cells and EPZ015666‐treated cells for each population (G1 vs not G1, S vs not S, G2/M vs not G2/M), in a Fisher‐exact test. We adjusted for multiple testing using the Benjamini‐Hochberg method. Differences were considered significant if the adjusted *P* value was below 0.05. For the in vivo experiment, differences observed between treated mice RTV and control group RTV were calculated using a two‐tailed Mann‐Whitney test. Differences were considered significant if the *P* value was below 0.05.

## 3 RESULTS

### 3.1 PRMT5 is differentially localized in TNBC compared to other breast cancer subtypes and to normal mammary tissues

**Figure 1:**
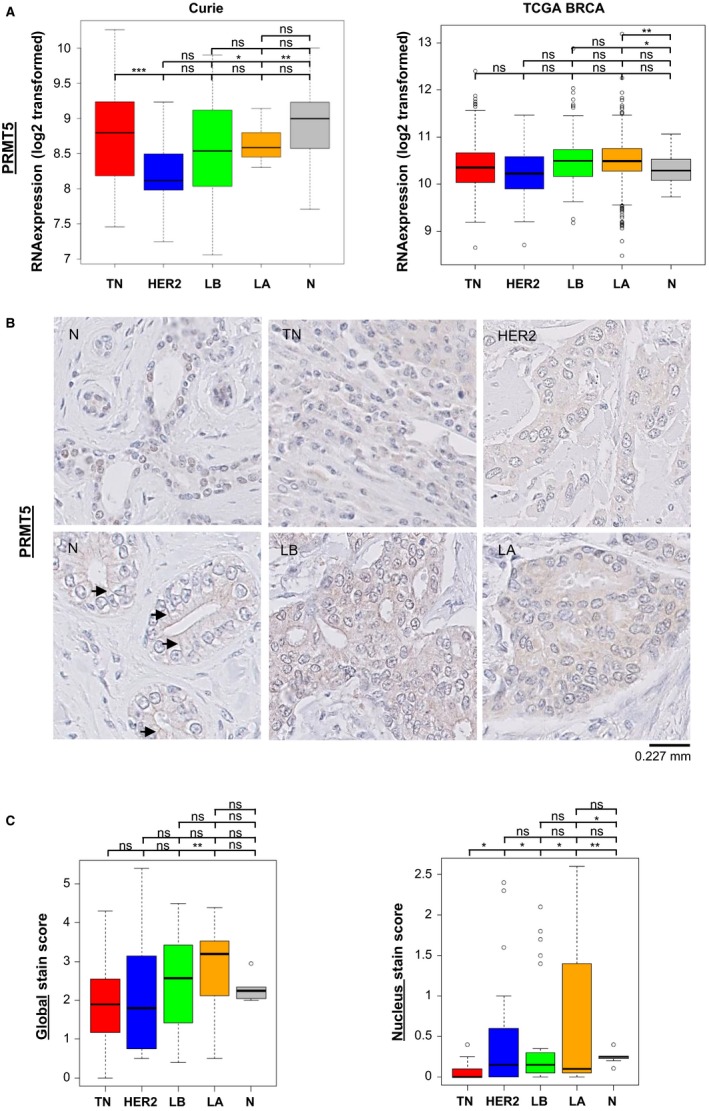
PRMT5 is differentially localized in breast cancer subtypes and healthy mammary tissues. A, PRMT5 mRNA expression in the different breast cancer subtypes and in normal breast tissues in the Curie^17^ (left panel) and TCGA BRCA^19^ (right panel) cohorts. The breast cancers subtypes rank from the most to the less proliferative tumors: TNBC (TN, red), ER‐/HER2+ (HER2, blue), luminal B (LB, green), luminal A (LA, orange). Normal breast tissues (N) are in grey. RNA relative quantifications are logarithmic (log2) transformed and illustrated by boxplots. Outliers are shown within each studied population (open circles). *P* values were calculated using ANOVA test and are indicated as follows: *P < 0.05, **P < 0.01, ***P < 0.001. B and C, PRMT5 protein levels were analyzed by immunohistochemistry (IHC) in the samples from the Curie cohort^17^: (B) Representative images of PRMT5 staining in the different breast cancer subtypes and in normal breast tissues (×40). Two images of normal breast tissues (N) are shown to better visualize nuclear (upper image) and transmembrane (bottom image) localization. Arrows indicate transmembrane staining. C, Global (left panel) and nuclear‐only (right panel) quantification of PRMT5 staining (0: no staining, 3: the strongest staining) in the different breast cancer subtypes (TN, red; HER2, blue; LB, green; LA, orange) and in normal breast tissues (N, grey). Boxplots show median, upper and lower quartiles of each studied population. Outliers are represented as open circles. *P* values were calculated using Student *t* test and are indicated as follows: *P < 0.05, **P < 0.01, ***P < 0.001

We examined the expression of PRMT5 in a previously generated cohort of 150 breast cancer biopsy specimens and normal breast tissues from the Institut Curie Hospital (Curie cohort).^17^ We find that TNBC express similar levels of PRMT5 mRNA compared to luminal breast cancers and healthy breast tissues, and higher levels of PRMT5 mRNA compared to HER2 + breast cancers (Figure 1A, left panel). To confirm these observations, we analyzed publically available data from the TCGA breast invasive carcinoma cohort.^19^ We find that PRMT5 is overexpressed in breast cancers—encompassing all subtypes—compared to normal breast tissues (data not shown), as previously reported.^25^ In contrast with the Curie cohort, the TCGA data shows no difference in PRMT5 mRNA expression between TNBC and HER2^+^ breast cancers (Figure 1A, right panel). However, Curie and TCGA cohort analyses indicate that TNBC, luminal breast tumors, and healthy breast tissues express comparable levels of PRMT5 mRNA (Figure 1A).

As mRNA and protein expression do not always concur, we next evaluated the expression of PRMT5 at the protein level in the samples from the Curie cohort by IHC using TMA, after validating an anti‐PRMT5 antibody for IHC staining (Figure S2). We observe that, as for mRNA, PRMT5 protein is expressed at similar levels in the different breast cancer subtypes and in healthy breast tissues (Figure 1B,C). However, the subcellular localization of PRMT5 varies (Figure 1B,C; Figure S3). Healthy breast tissues display significantly high levels of PRMT5 at the cell plasma membrane compared to cancerous tissues from all breast cancer subtypes (Figure 1B; Figure S3, left panel). Importantly, TNBC exhibit a distinctive PRMT5 subcellular distribution, with significantly lower levels of nuclear PRMT5 than healthy breast tissues and all other breast cancer subtypes (Figure 1C).

### 3.2 High PRMT5 expression is associated with poor prognosis in TNBC

**Figure 2:**
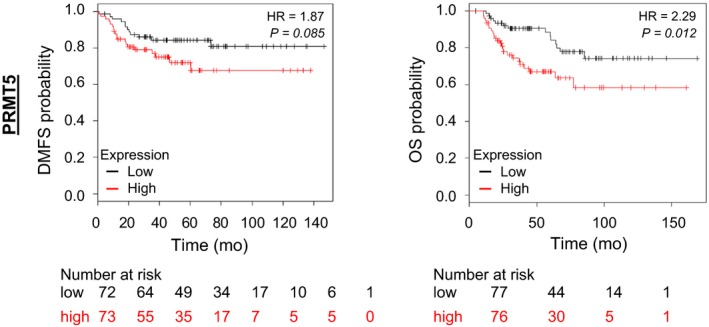
High PRMT5 expression associates with poor prognosis in TNBC. Distant metastasis free survival (DMFS, left panel) and overall survival (OS, right panel) according the RNA expression of PRMT5 (Affy probe ID: 1564520_s_at) was analyzed by Kaplan‐Meier (KM) Plotter^52^ (http://kmplot.com). Because the breast cancer subtypes have different prognoses, the analysis was restricted to TNBC patients: the group “basal” (ER‐/HER2‐) was selected from the intrinsic subtypes. TNBC samples were split into high and low groups according to the expression level of the selected probe (median cutoff). Hazard ratio with 95% confidence interval and log rank P value were calculated and significance threshold was set at *P* < 0.05. A similar figure of TNBC patient OS as a function of high vs low PRMT5 RNA expression is presented in Wu Y et al,^25^ with a lower number of samples (248 in our study compared to 220 in that article)

Our analysis of PRMT5 mRNA expression in TNBC shows up to eightfold variability between samples (Figure 1A), paralleling TNBC heterogeneity.^2^ To determine the clinical significance of PRMT5 in TNBC, we analyzed PRMT5 expression and survival outcomes in TNBC using data from the Kaplan‐Meier plotter online database^26^ (www.kmplot.com) (Figure 2). Kaplan‐Meier analyses indicate an association between high PRMT5 expression and lower probabilities of distant metastasis‐free survival (DMFS, *P* = 0.085) and overall survival (OS, *P* = 0.012) (Figure 2), outlining the potential therapeutic value of PRMT5 targeting in a subset of TNBC.

### 3.3 Pharmacological inhibition of PRMT5 impairs breast cancer cell viability

To explore the potential of PRMT5 targeting in TNBC, we first examined the effect of PRMT5 depletion on six TNBC cell lines using two PRMT5 siRNAs. PRMT5 silencing significantly decreases viability (Figure S4A), and colony formation (Figure S4B) of all tested cell lines.

**Figure 3:**
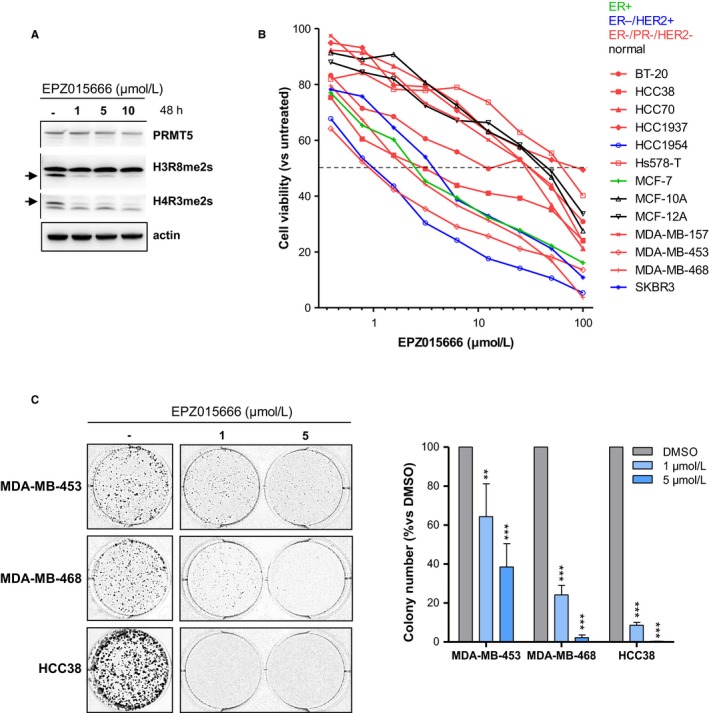
PRMT5 inhibition impairs cell viability. A, EPZ015666 inhibits PRMT5 activity. MDA‐MB‐468 cells were treated with the indicated concentration of the PRMT5 inhibitor EPZ015666 or with vehicle (DMSO). PRMT5 activity was assessed 48 h later by Western‐Blot analysis using antibodies that recognize symmetric dimethyl‐arginine on histones H3 (H3R8me2s) and H4 (H4R3me2s). PRMT5 expression was verified. Actin was used as a loading control. Images are from a single experiment representative of three independent experiments. B, Treatment of breast cell lines with PRMT5 inhibitor EPZ015666 identifies a group of sensitive cell lines and a group of resistant cell lines. Cell viability was determined by MTT assay after four doubling times. Results are expressed as the percentage of cell growth relative to vehicle‐treated cells. The mean of at least three independent experiments for each cell line is presented. Breast cancer subtypes are indicated as follows: green (ER+), blue (ER‐/HER2+), red (ER‐/PR‐/HER2‐). The non‐tumorigenic breast cells, MCF‐10A and MCF‐12A, are in black. C, PRMT5 inhibition reduces colony formation. MDA‐MB‐453, MDA‐MB‐468 and HCC38 TNBC cells, seeded at low‐confluency, were treated with DMSO (‐) or with 1 or 5 µmol/L EPZ015666 for 9‐14 d, until colony formation. A representative image of one well is shown for all conditions (left panel). The number of colonies, counted using ImageJ Software (NIH) is presented as a percentage relative to DMSO‐treated cells (right panel). Grey bars: DMSO‐treated cells; blue bars: EPZ015666‐treated cells. Represented are means + SD from at least three independent experiments. *P* values were calculated using Student *t* test and are indicated as follow: **P < 0.01, ***P < 0.001 (ie decrease relative to the control DMSO)

To better assess the therapeutic relevance of PRMT5 targeting in TNBC, we next examined the effect of PRMT5 inhibition on a panel of breast cell lines using the PRMT5‐specific inhibitor EPZ015666.^13^, ^14^, ^27^ First, we confirmed that EPZ015666 inhibits PRMT5 activity by analyzing PRMT5‐specific methylation marks on histones H3 (H3R8me2s) and H4 (H4R3me2s) (Figure 3A; Figure S5). We then conducted a cell viability assay on 13 breast cell lines, comprising eight TNBC‐derived cell lines (ER‐/PR‐/HER‐) from the different TNBC molecular subtypes defined by Lehmann^2^ (Table S3), but also one ER+ (MCF‐7), two ER−/HER2+ (HCC1954, SKBR3), and two non‐tumorigenic mammary cell lines (MCF‐10A, MCF‐12A), for comparison purposes. EPZ015666 treatment impairs cell viability of all tested cell lines (Figure 3B). More precisely, we distinguish two groups of cell lines that we deem “sensitive” or “resistant” to the inhibitor based on the IC50 values calculated from the assay (0.5 µmol/L < IC50 < 4 µmol/L and IC50 >30 µmol/L, respectively) (Figure 3B, Table S3). Three of the eight TNBC (ER−/PR−/HER−) cell lines tested are sensitive. These three cell lines—MDA‐MB‐453, MDA‐MB‐468, and HCC38—are among the four most sensitive to the PRMT5 inhibitor and therefore represent good models to study the impact of PRMT5 inhibition on TNBC. The two HER2+ cell lines (HCC1954, SKBR3) and the single luminal cell line (MCF‐7) tested are sensitive. The non‐tumorigenic mammary cell lines MCF‐10A and MCF‐12A are resistant (Figure 3B). This differential sensitivity to EPZ015666 is not due to marked differences in PRMT5 expression nor in global PRMT5 activity (Figure S6).

A newer more potent PRMT5 inhibitor (biochemical IC50 of 6.2 ± 0.8 nmol/L^16^ vs 22 ± 14 nmol/L for EPZ015666^27^), GSK3326595 (EPZ015938), is currently evaluated in a phase I clinical trial.^16^, ^28^ In order to confirm the specificity of EPZ015666, we examined its effect on four TNBC cell lines—two sensitive (MDA‐MB‐453, MDA‐MB‐468) and two resistant (BT‐20, HCC70) to EPZ015666. We first validated the inhibition of PRMT5 activity by EPZ015938 in the four cell lines (Figure S7A). Like EPZ015666, EPZ015938 impairs the viability of MDA‐MB‐453 and MDA‐MB‐468 cells (Figure S7B), but with more efficacy. Indeed, we calculate IC50 values of 124 nmol/L and 162 nmol/L for MDA‐MB‐453 and MDA‐MB‐468 cells, respectively (vs 1 µmol/L and 2.2 µmol/L for EPZ015666). BT‐20 and HCC70 cells are resistant to EPZ015938 (IC50 >35 µmol/L for both cell lines) (Figure S7A), like to EPZ015666 (Figure 3B, Table S3).

### 3.4 PRMT5 inhibition impairs colony formation in TNBC cells

We pursued our study by investigating the molecular mechanisms of the PRMT5‐dependent cell survival in the three TNBC cell lines sensitive to EPZ015666: MDA‐MB‐453, MDA‐MB‐468, and HCC38.

To further validate the deleterious effect of PRMT5 inhibition on cell viability (Figure 3B), we examined the effect of PRMT5 inhibition on colony formation. In the three tested cell lines, 1 µmol/L EPZ015666 treatment results in 35%‐90% less colonies (Figure 3C) compared to untreated cells. EPZ015666 treatment at 5µmol/L results in 60%‐100% less colonies (Figure 3C).

### 3.5 PRMT5 inhibition induces apoptosis and G2/M cell cycle arrest

**Figure 4:**
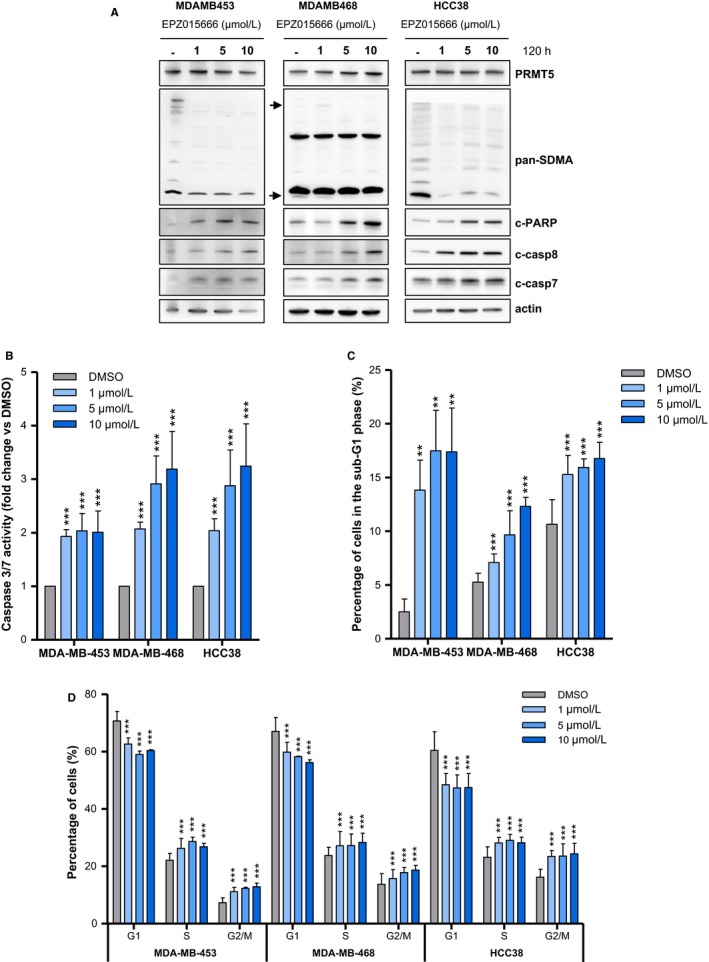
PRMT5 inhibition leads to apoptosis and affects cell cycle progression. A‐D, MDA‐MB‐453, MDA‐MB‐468 and HCC38 TNBC cells were treated with DMSO (‐ or grey bars) or with the indicated concentration of EPZ015666 (1‐10 µmol/L, blue bars) (A‐C) PRMT5 inhibition induces apoptosis. A, Apoptosis was analyzed by western blotting using antibodies that recognize the cleaved forms of caspase 7 (c‐casp7), caspase 8 (c‐casp8) and PARP (c‐PARP) 120 h after PRMT5 inhibition. PRMT5 and actin were used as controls. General symmetric arginine dimethylation (pan‐SDMA) was examined to validate PRMT5 inhibition following cell treatment with EPZ015666. Pictures are from a single experiment representative of two or three independent experiments. B, Apoptosis was assessed by a luminescence assay to detect caspase 3/7 activity of viable cells 120h after PRMT5 inhibition. Results are expressed as fold‐change compared to vehicle‐treated cells. *P* values were calculated using Student *t* test. C and D, Cell cycle was monitored 96 hours following PRMT5 inhibition or treatment with DMSO by FACs analysis following PI staining. C, Percentage of cells in the sub‐G1 phase are represented. Data are expressed as means from three to four independent experiments. *P* values were calculated using Student *t* test. D, PRMT5 inhibition impairs cell cycle progression. Percentages of live cells in G1, S and G2/M phases are represented. *P* values were calculated based on cell count using a Fisher‐exact test and adjusted for multiple testing using the Benjamini‐Hochberg method. B‐D, Means + SD of at least three independent experiments are represented. *P* values (B and C) and adjusted *P* values (D) are indicated as follows: **P < 0.01; ***P < 0.001

We first evaluated the activation of apoptotic pathways in EPZ015666‐treated cells. Western blot analyses confirm PRMT5 inhibition (decreased pan‐SDMA), and show dose‐dependent increases in PARP, caspase‐7, and caspase‐8 cleavage following treatment (Figure 4A). These results are supported by the detection of increased caspase‐3 and caspase‐7 activity in a luminescent assay (Figure 4B).

Next, we examined the effect of PRMT5 inhibition on breast cancer cell cycle progression using flow cytometry. EPZ015666‐treated MDA‐MB‐453, MDA‐MB‐468, and HCC38 cells display a higher proportion of cells in the sub‐G1 phase 96 hours post‐treatment compared to untreated cells (Figure 4 C), confirming the above results regarding apoptosis. In all three cell lines, we also observe a significant decrease in the G1 population and an increase of the G2/M population following EPZ015666 treatment (Figure 4D). Collectively, our data show that PRMT5 inhibition induces apoptosis and impedes cell cycle progression.

### 3.6 PRMT5 inhibition impairs mammosphere formation in TNBC cells

**Figure 5:**
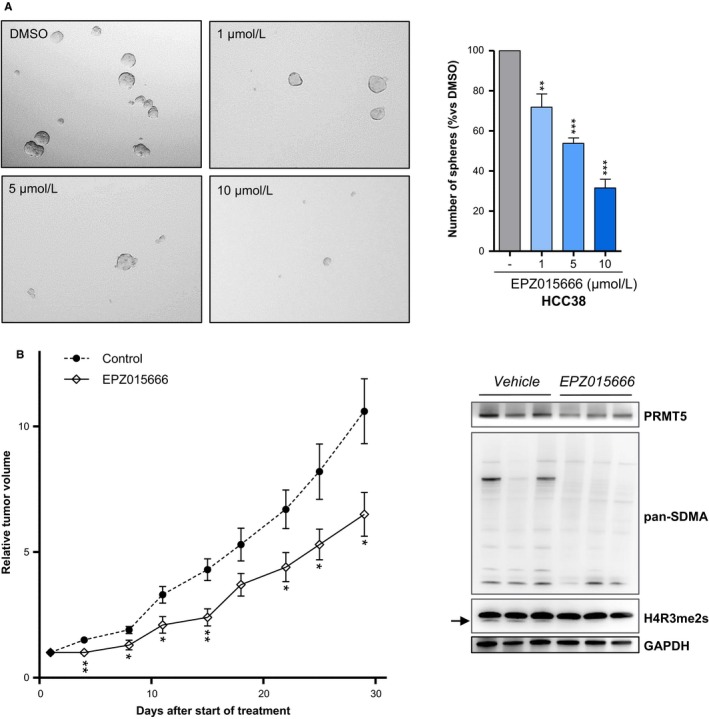
PRMT5 inhibition impairs, mammosphere formation, and slows tumor growth in a TNBC PDX model. A, PRMT5 inhibition impairs mammosphere formation. HCC38 cells were seeded in 6‐well low‐binding plates and treated with DMSO (‐) or with the indicated concentration of EPZ015666 for 14 d. Mammospheres were then examined and counted under a microscope. A representative image of one well is also shown for all conditions (×100). Mammosphere count for each EPZ015666‐treated condition is expressed as a percentage relative to vehicle‐treated cells. Grey bars: DMSO‐treated cells; blue bars: EPZ015666‐treated cells. Represented are means + SD from at least three independent experiments. *P* values were calculated using Student *t* test and are indicated as follows: *P < 0.05, **P < 0.01, ***P < 0.001 (ie, decrease relative to the control DMSO). B, PRMT5 inhibition slows tumor growth in vivo. EPZ015666 (200 mg/kg BID, *po*) was administered to a TNBC PDX model (n = 7 mice) during one month. Control mice were treated with EPZ015666 vehicle (n = 7 mice). (left panel) Tumor volume was measured twice weekly with calipers. Growth curves were obtained by plotting relative tumor volume mean versus time ± SEM *P* values were calculated using Mann‐Whitney test are indicated as follow: *P < 0.05, **P < 0.01, ***P < 0.001. (right panel) PRMT5 inhibition reduces symmetric arginine dimethylation (SDMA) in tumors. Western blot analysis of tumors at the end of the treatment shows lower PRMT5 activity in the tumors derived from EPZ015666‐treated mice compared to those derived from vehicle‐treated mice. Symmetric arginine dimethylation was detected using anti‐pan‐SDMA and anti‐H3R8me2s antibodies. PRMT5 expression was verified. GAPDH was used as loading control

In addition, because breast cancer stem cells (BCSCs) are enriched in TNBC^8^ and play a role in resistance to chemotherapies,^29^ we investigated the effect of PRMT5 inhibition on an indicator of breast cancer cell stemness. Specifically, we assessed the propensity of HCC38 cells to form mammospheres following EPZ015666 treatment. PRMT5 inhibition significantly impairs HCC38 mammosphere formation in a dose‐dependent manner (Figure 5A), suggesting a potential role for PRMT5 in the maintenance of BCSC properties.

### 3.7 PRMT5 targeting slows tumor progression in vivo in a TNBC patient‐derived xenograft model

We evaluated the potential anti‐tumor effects of PRMT5 targeting in a preclinical study involving a TNBC patient‐derived xenograft model, selected for its high PRMT5 mRNA expression. EPZ015666 was administered twice daily, at 100 mg/kg *per‐os* (*po*). Treatment significantly slows tumor growth, with 39% tumor growth inhibition (TGI, *P* = 0.02) after 4 weeks (Figure 5B, left panel), and no observed toxicity (Figure S1). We verified that PRMT5 inhibition had indeed occurred in the tumors at the end of the experiment by assessing PRMT5 activity by Western Blot, using pan symmetric dimethyl‐arginine (pan‐SDMA) and H4R3me2s antibodies (Figure 5B, right panel).

### 3.8 Synergistic interaction between PRMT5 and EGFR inhibitors

**Figure 6:**
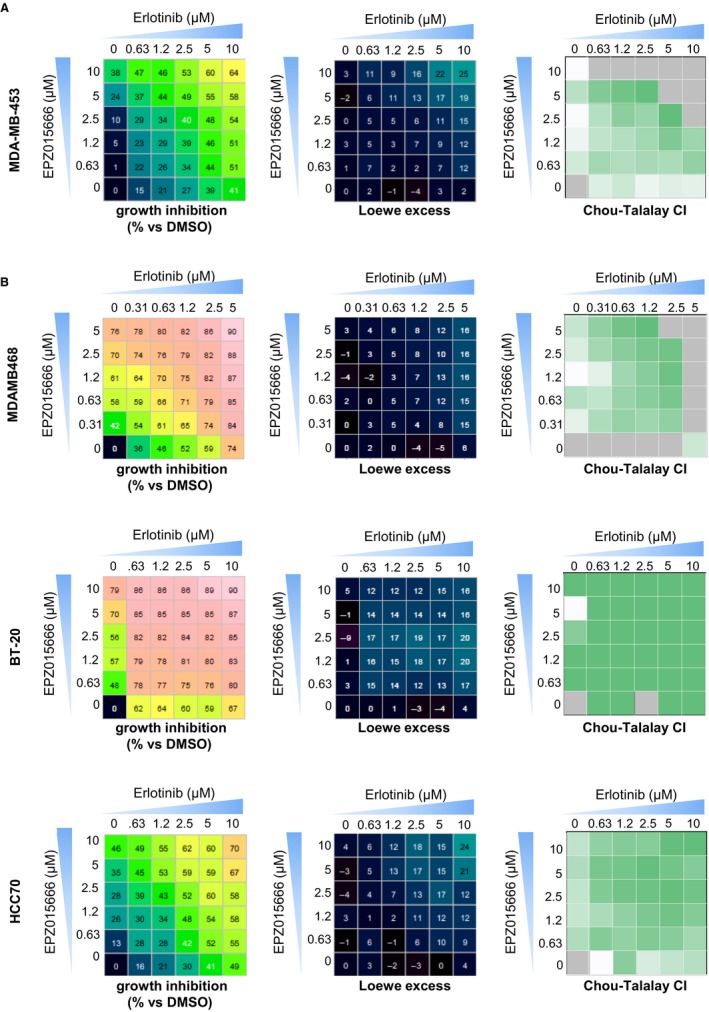
Synergistic interaction between PRMT5 and EGFR inhibition. A and B, Potentiation between EPZ015666 and Erlotinib. Cells were seeded into 96‐well plates and treated with EPZ01566 and/or Erlotinib for 7 d at 5 ‐twofold‐serial diluted concentrations (excluding a drug‐less control; concentrations as indicated). Cell viability was measured after 3 d, as in the drug combination screen (A), or 7 d (B) using CellTiter‐Glo (CTG). The nature of the interaction between EPZ015666 and Erlotinib was assessed by Loewe excess and Chou‐Talalay combination index (CI). Cell viability inhibition (% compared to DMSO‐treated cells) is indicated for each combination (left panel, dose matrix). Chalice Analyzer (http://chalice.horizondiscovery.com/analyzer-server/cwr/analyze.jsp) was used to calculate the Loewe excess (middle panel). Synerdrug Analyzer (https://github.com/bioinfo-pf-curie/synerdrug) was used to calculate Chou‐Talalay CIs (right panel). CIs are not calculated where the effect of the drug combination is superior to that of either drug alone (grey quadrants). Data are representative of at least three independent experiments

Drug combinations have gained interest in cancer therapeutics, as means for increased treatment efficacy, decreased toxicity, and reduced risk of drug resistance. To address this, we screened the Prestwick Chemical Library—consisting of 1,200 FDA‐approved molecules^30^—and six additional drugs (Table S2), alone or in combination with EPZ015666 on MDA‐MB‐453 cell viability. Following the selection criteria, Erlotinib, an EGFR inhibitor, was identified twice in the top 25 compounds for which there is at least some additivity with EPZ015666 (Figure S8). We validated the results of our screen by treating MDA‐MB‐453 cells with variable combinations of EPZ015666 and Erlotinib and measuring cell viability after 3 days, as for the screen (Figure 5, left panel). We calculated Loewe excess inhibition values and Chou‐Talalay combination indexes (CI) as measures of synergy (Figure 5, middle and right panels, respectively). We considered Loewe excess values greater than 10% and Chou‐Talalay CI lower than 1 to be suggestive of additivity. Both Loewe excess and Chou‐Talalay CI suggest additivity, if not synergy, between EPZ015666 and Erlotinib, confirming the results from our screen (Figure 6A). We next sought to determine whether an EPZ015666/Erlotinib drug combination would be efficient on all TNBC cell lines or, as for EPZ015666 treatment alone, in a subset of TNBC cell lines. We hence tested combinations of EPZ015666 and Erlotinib on four additional cell lines, two of which (MDA‐MB‐468, HCC38) were sensitive to EPZ015666 in our initial cell viability assay (Figure 3A), and two of which (BT20, HCC70) were resistant. We found the drug combination to be beneficial on MDA‐MB‐468 and BT20 cells especially (Figure 6B), both of which express high levels of EGFR (Figure S5B), as well as on HCC70 cells (Figure 6B). EPZ015666/Erlotinib combination had no additive effect on HCC38 cells (data not shown).

## 4 DISCUSSION

Despite considerable improvement in breast cancer therapeutic management, no targeted therapy yet exists for the treatment of TNBC, and this breast cancer subtype remains a challenge for oncologists. PRMTs have recently received considerable attention as potential therapeutic targets in various types of cancer,^31^ and several specific PRMT inhibitors have recently been described.^14^ The present study suggests a promising therapeutic potential for PRMT5 targeting in a subset of TNBC, using the small‐molecule inhibitor EPZ015666.^13^, ^14^, ^27^ Indeed, we show that PRMT5 inhibition (a) impairs breast cancer cell viability, (b) triggers apoptosis, (c) impedes colony formation (d) affects CSC properties, and (e) slows tumor growth in a TNBC patient‐derived xenograft model (PDX). In doing so, we align with previous studies which underline the potential value of PRMT5 inhibition as a therapeutic approach in glioblastoma^32^, ^33^ and mantle cell lymphoma.^8^, ^13^

The 13 breast cell lines we tested display differential sensitivity to PRMT5 inhibition. Six of the cell lines examined are sensitive whereas seven are resistant to EPZ015666. Interestingly, the non‐tumorigenic cell lines MCF‐10A and MCF‐12A belong to the group that is resistant to PRMT5 inhibition. Although non‐tumorigenic, these cell lines are the most proliferative in our in vitro assays, demonstrating that the sensitivity to the inhibitor is not related to cell proliferation rate. This consideration suggests that side‐effects of PRMT5 targeting could be minimal. We confirmed specificity and differential sensitivity to PRMT5 inhibition using a newly available inhibitor, EPZ015938. Using this same inhibitor, Gerhart et al also observe variable sensitivity to PRMT5 inhibition across a panel of 240 cancer cell lines.^16^ They find p53 status to be determinant to EPZ015938 sensitivity. We, however, observe no correlation between p53 mutation and sensitivity to PRMT5 inhibition in the TNBC cell lines we tested, and were unable to trace sensitivity back to the expression of transcriptomic biomarkers. Enlarging our analysis to a larger number of TNBC cell lines would be necessary to do so, and could, in turn, help stratify patients who could benefit from treatment with a PRMT5 inhibitor.

In contrast, PRMT5 depletion impairs the viability of all the TNBC cell lines tested in this study, thus aligning with previous research conducted on cell lines derived from other cancer types^10^, ^34^ or on MDA‐MB‐468^16^ and another TNBC‐derived cell line, MDA‐MB‐231.^8^, ^25^ It thus appears that the effect of PRMT5 depletion on cell viability is independent from cell sensitivity to EPZ015666. Indeed, inhibiting an enzyme is different from removing its expression. Such a difference was also observed by Mavrakis et al,^35^ who found that cancer cell sensitivity to PRMT5 depletion, but not to PRMT5 inhibition using EPZ015666, is contingent upon low‐MTAP expression. These observations demonstrate that inhibiting the activity of PRMT5 has a different impact on cell viability than silencing PRMT5 expression. The mode of PRMT5 targeting is therefore key. More generally, these observations underline the importance of validating potential therapeutic targets using pharmacological inhibitors, and not only using siRNA.

Furthermore, we find that PRMT5 is required for cell proliferation. Pharmacological inhibition of PRMT5 slows cell cycling, leading towards a G2/M cell cycle arrest in the three TNBC cell lines examined. G2/M arrest was previously observed in U‐87 MG human glioma cells^36^ treated with EPZ015666, and in NIH‐3T3 cells stably expressing an anti‐sense PRMT5.^9^ Some studies have shown that following PRMT5 knockdown, Huh7, MCF‐7, and MDA‐MB‐231 cells also exhibit decreased proliferation, but associated to a G1/S growth arrest.^12^, ^25^, ^37^ The mode of PRMT5 targeting is likely determinant here. It is also possible that the role of PRMT5 in cell cycle progression be dependent on cell‐type and context.

Previous studies demonstrate that PRMT5 activity is essential for cell stemness.^7^, ^38^, ^39^ In breast cancers specifically, PRMT5 was shown to play a critical role in the proliferation and self‐renewal of stem‐like cells via the regulation of C‐MYC, OCT4/A, and FOXP1 expression.^7^, ^38^, ^39^ Our study supports these findings as we show that PRMT5 inhibition impairs the formation of mammospheres—an indicator of cancer cell stemness—in a TNBC cell line. Since TNBC are enriched in stem‐like cells, and this subpopulation is linked to resistance to chemotherapy and relapse,^29^ our observations further support the coherence of targeting PRMT5 in TNBC and suggest that PRMT5 targeting could potentiate the effects of conventional therapy, potentially avoiding relapses, the main concern for current treatments of TNBC patients.

Our screening of the Prestwick Chemical Library in the presence or in the absence of the PRMT5 inhibitor EPZ015666 reveals that targeting EGFR potentiates the effect of PRMT5 inhibition on cell viability. In four TNBC cell lines, we thus show potentiation between Erlotinib and EPZ015666 using Loewe excess quantification and Chou‐Talalay combination index. TNBC cell line sensitivity to the drug combination was independent from sensitivity to EPZ015666 treatment alone, suggesting that PRMT5 targeting may be valuable on a different set—and perhaps wider number—of TNBC when used in combination than when used as monotherapy. In our cell line panel, we noted that the EPZ015666/Erlotinib combination is most effective on, but not limited to, the two cell lines expressing high levels of EGFR. EGFR inhibitors on their own have shown only a modest effect in clinical trials in TNBC patients,^40^ their use in combination could be beneficial. PRMT5 has been shown to interact with EGFR and to modulate its activity.^41^ EGFR is also a substrate of PRMT1,^42^ the main PRMT generating asymmetric dimethylation.^31^ The methylation of EGFR by PRMT1 is reported to play a role in resistance to treatment with Cetuximab, an anti‐EGFR antibody.^42^ Recently, inhibition of PRMT1 using the nonspecific inhibitor Furamidine was reported to increase Erlotinib sensitivity in MDA‐MB‐468 cells,^43^ further endorsing anti‐EGFR/anti‐PRMT therapeutic combination strategies in the context EGFR‐overexpressing TNBC. Previous reports have shown that PRMT5 silencing slows tumor growth in vivo, using xenograft models from cancer‐derived cell lines, including the breast cancer‐derived MCF7.^39^, ^44^ In alignment with these reports, we here show that pharmacological inhibition of PRMT5 slows tumor growth in a TNBC PDX model. Our study constitutes a first approach to PRMT5 inhibition in vivo in TNBC PDX models. Applying this approach to additional PDX models would be essential to strengthen this initial observation, as well as to evaluate in vivo the potential of EGFR and PRMT5 combinatorial targeting.

Parallelly, using Kaplan‐Meier plotter online survival analyses in TNBC, we associate the high PRMT5 expression and poor patient prognosis in TNBC, as observed in a wide range of cancers (Stopa, Krebs, and Shechter 2015, 2041‐2059), including breast cancers.^25^, ^45^ We do not, however, observe elevated PRMT5 mRNA expression in TNBC from our breast cancer cohort nor from TCGA‐BRCA. In our cohort, PRMT5 is not overexpressed at the protein level either, contrarily to reports from distinct groups.^45^, ^46^ These discrepancies may be due to the use of different TMA fixation and staining techniques, as well as to the use of different PRMT5 antibodies.

We further show here that PRMT5 is differentially localized in breast cancers and healthy mammary tissue, and that some important subcellular localization differences can be noted between breast cancer subtypes. Indeed, PRMT5 is expressed at lower levels in the nucleus of TNBC than in those of healthy breast tissues, HER2+, and luminal breast cancers. We thus posit that PRMT5 activity has different biological outcomes depending on PRMT5 subcellular localization and/or substrate specificity.

Several studies have already pointed out the importance of PRMT5 localization in determining substrate specificity and resultant cell fate. During mouse embryogenesis, Prmt5 is predominantly found in the cytoplasm, where it maintains cell pluripotency via methylation of predeposited H2AR3.^7^ The onset of cell differentiation is contingent on the nuclear translocation of Prmt5 and concurrent decrease of the H2AR3 methylation mark.^7^ Likewise, PRMT5 localizes in the cytoplasm of human prostate cancer cells where it supports cell proliferation.^47^, ^48^ Forced nuclear localization of PRMT5 is associated with epithelial cell differentiation and inhibits prostate cancer development in tissue culture and in prostate tumor xenograft models.^49^ More recently, Lattouf et al found that in a cohort of 390 breast invasive carcinomas, high nuclear PRMT5 was associated with longer OS and longer DFS, thus concurring with our study.^50^ Broadly, the majority of cancer‐related studies interested in PRMT5 localization and substrate specificity associates cytoplasmic PRMT5 activity to tumor development or bad prognosis. We further this observation by suggesting that the localization of PRMT5 may be a determinant of TNBC.

What controls the subcellular localization of PRMT5? A 2007 study by Teng et al may give a first element of response.^51^ In this study, Teng et al show that treatment of prostate cancer cells using the nucleolin octamer AS1411, leads to a nucleolin‐mediated redistribution of PRMT5 from the nucleus to the cytoplasm. Teng et al thus posit that nucleolin plays a role in PRMT5 shuttling between these subcellular compartments. It is likely, however, that the mechanism proposed by Teng et al not be the sole at work, and that other PRMT5 partners may be involved in its nuclear/cytosolic shutting.

Which additional signals and/or protein‐protein interactions are involved? These interrogations must be addressed to better understand PRMT5 substrate specificity and to further decipher the role of PRMT5 in cancers. Such knowledge could, in turn, allow the design of efficient therapeutic strategies targeting PRMT5 in a substrate‐specific manner. In conclusion, the present study highlights the importance of the subcellular localization of PRMT5 in determining TNBC prognosis and upholds continued attention for PRMT5 targeting, alone or in combination, as a potential treatment option in a subset of TNBC.
