# Supplementary material for: Protein arginine methyltransferase 5: A novel therapeutic target for triple‐negative breast cancers
Source: Cancer Med. 2019 Apr 8;8(5):2414–28. doi: 10.1002/cam4.2114 (PMC6537044; doi:10.1002/cam4.2114)
Supplement: Supplementary file 1 [file CAM4-8-2414-s001.pdf]

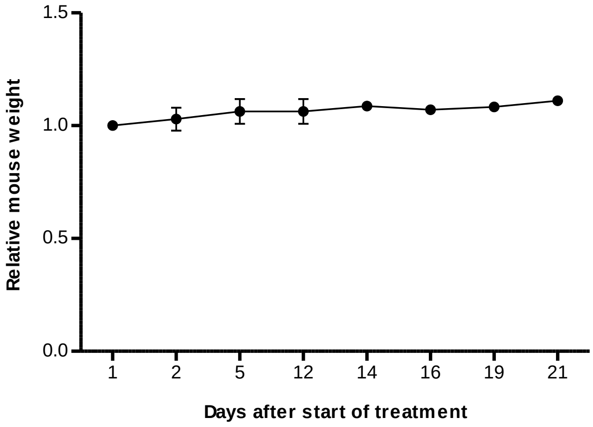

Supplementary Figure S1. EPZ015666 shows no toxicity for mice.

Nude mice (n=3) were administered EPZ015666 at 100 mg/kg *per-os* (*p.o.*), twice daily. Treatment was not associated with any mortality or body weight loss over the 21-day experimental period.

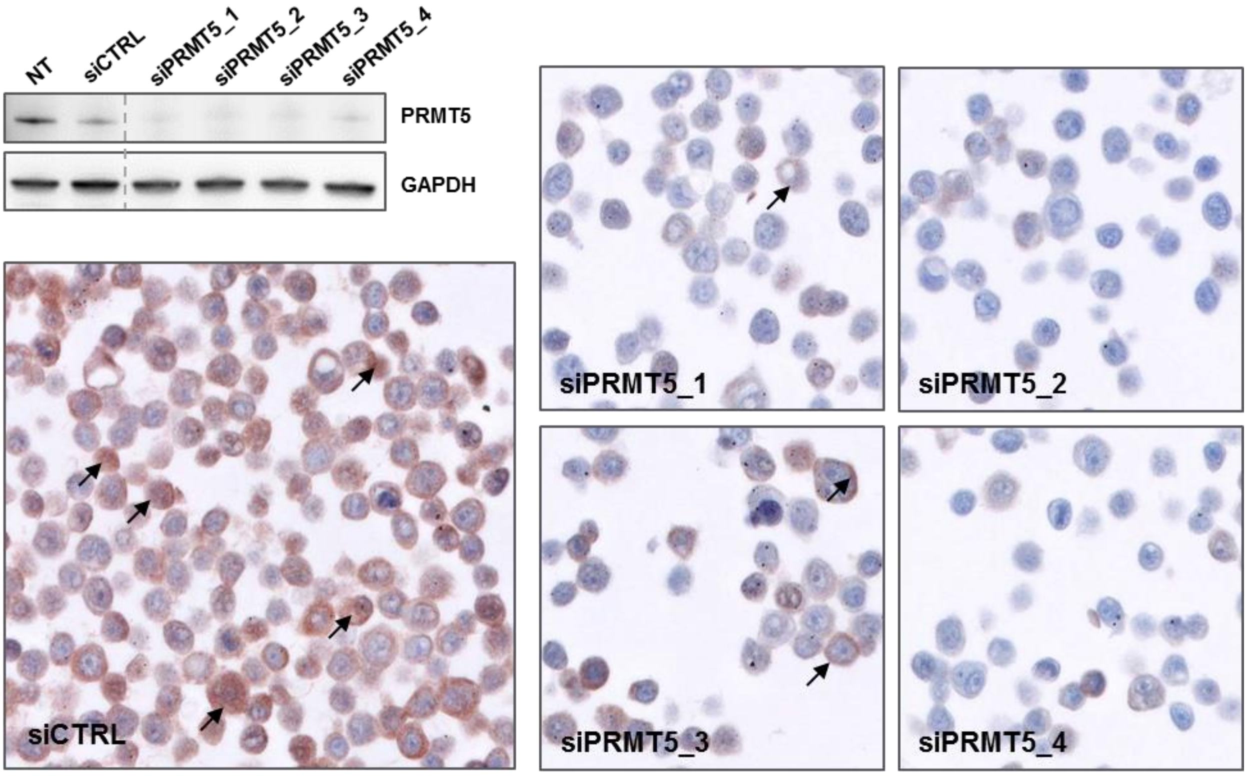

Supplementary Figure S2. PRMT5 antibody validation for IHC staining.

MDA-MB-468 cells were transfected with 20nM siRNA targeted against PRMT5 or with a control siRNA (siCTRL), or left untreated (NT). PRMT5 expression was assessed 72 hours later by western blotting. GAPDH was used as loading control. Part of the cells were collected, pelleted, frozen and fixed for IHC staining, as previously described[17,19].

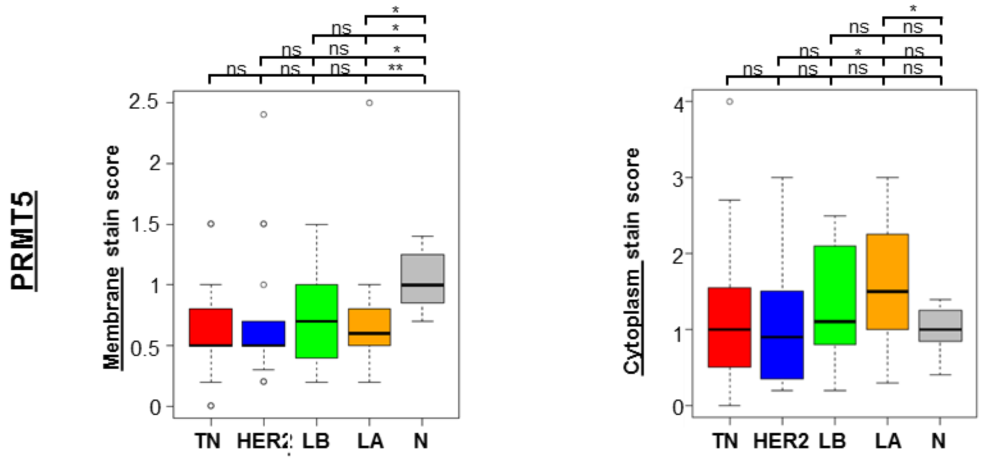

Supplementary Figure S3. Subcellular localization of PRMT5 in the Curie cohort.

PRMT5 protein levels were analyzed by immunohistochemistry (IHC) in the samples from the Curie cohort[16]: TNBC (TN, red), $ER^-/HER2^+$ (HER2, blue), luminal B (LB, green), luminal A (LA, orange), and normal breast tissues (N, grey). Staining was quantified (0: no staining, 3: the strongest staining) at the cell membrane (left panels), and in the cytoplasm (right panels). Boxplots show median, upper and lower quartiles of each studied population. Outliers are represented as open circles. *P* values were calculated using Student t-test and are indicated as follows: *$P<0.05$, **$P<0.01$, ***$P<0.001$.

a

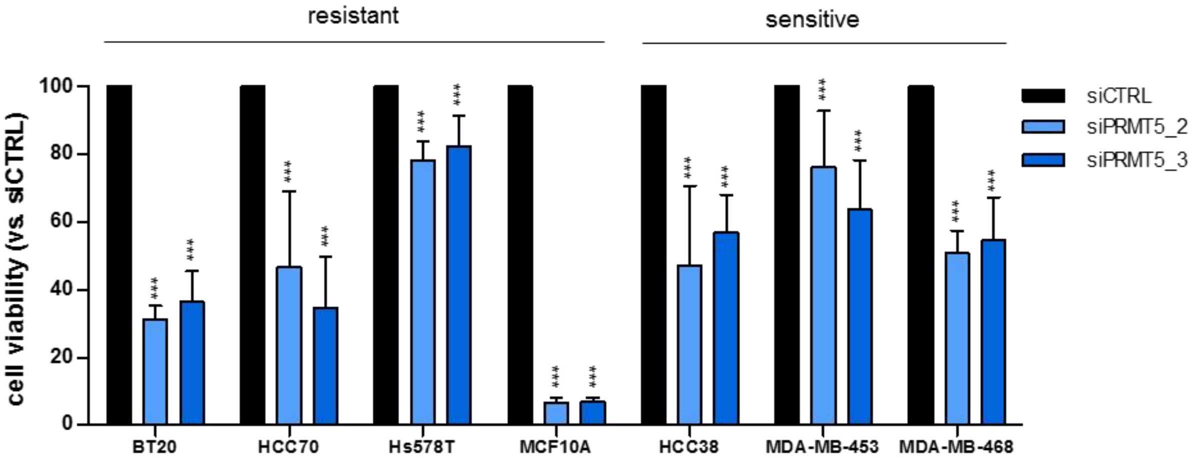

resistant
sensitive
cell viability (vs. siCTRL)
100
80
60
40
20
0
BT20
HCC70
Hs578T
MCF10A
HCC38
MDA-MB-453
MDA-MB-468
siCTRL
siPRMT5_2
siPRMT5_3

b

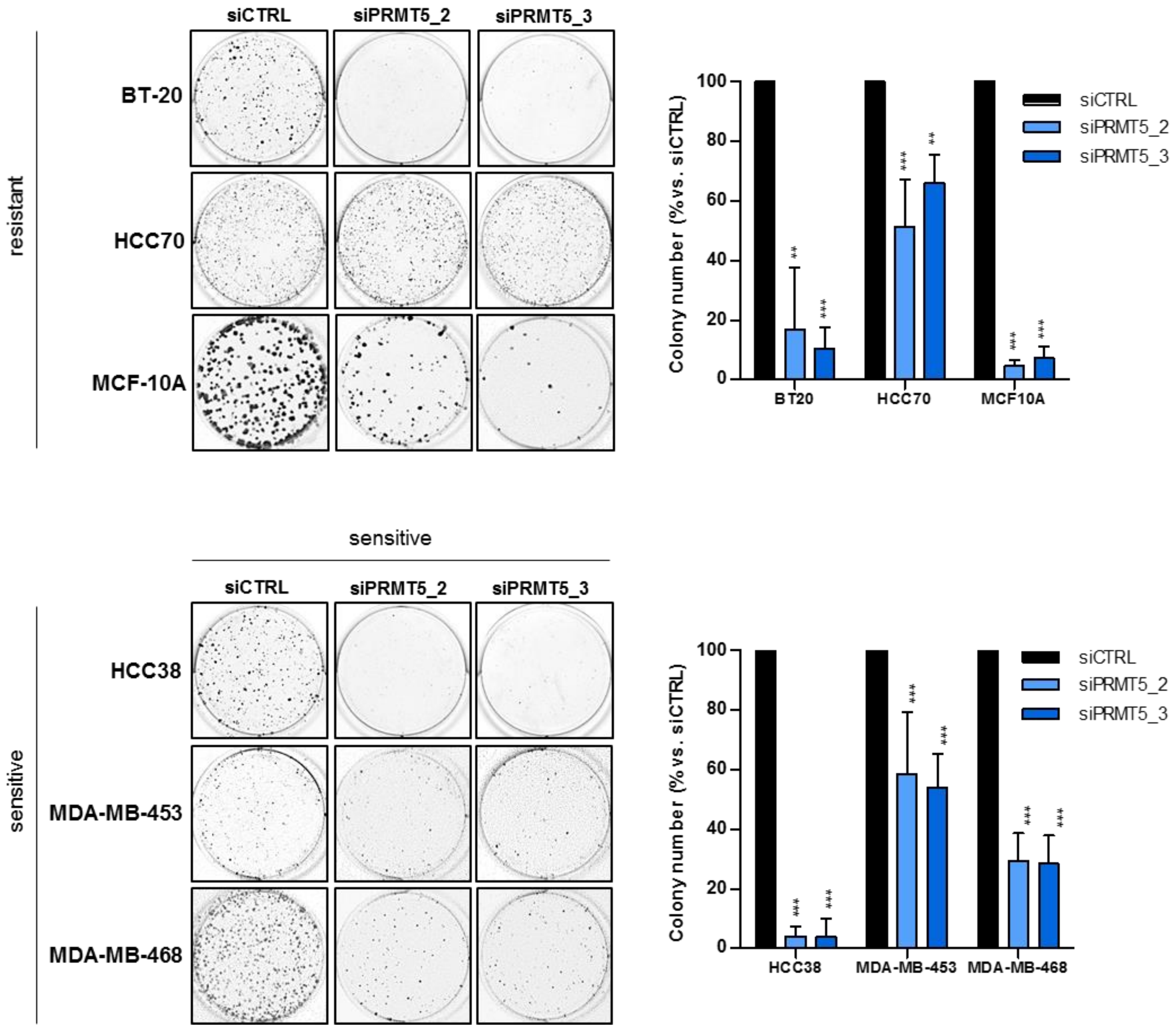

siCTRL
siPRMT5_2
siPRMT5_3
resistant
BT-20
HCC70
MCF-10A
Colony number (% vs. siCTRL)
BT20
HCC70
MCF10A
sensitive
HCC38
MDA-MB-453
MDA-MB-468

Supplementary Figure S4. PRMT5 depletion impairs TNBC cell viability and clonogenicity. (a) Cells were transfected with one of two PRMT5 siRNAs (PRMT5_2, PRMT5_3), chosen for their high efficacy, or control siRNA (siCTRL). Depletion was validated in all cell lines, and cell viability was determined by MTT assay after 6 days. Results are expressed as the percentage of cell growth relative to siCTRL-transfected cells. Presented is the mean of at least three independent experiments for each cell line. Cell lines are distributed according to their sensitivity to PRMT5 inhibitor EPZ015666. (*b*) TNBC cells were transfected with the indicated siRNA and seeded at low confluency for 5-14 days, until colony formation. A representative image of one well is shown for all conditions (left panel) Average colony number was evaluated using ImageJ Software (NIH) and is represented as a percentage relative to siCTRL-transfected cells. Represented are means + SD from at least three independent experiments (right panel). Black bars: siCTRL-transfected cells; blue bars: siPRMT5-transfected cells. *P* values were calculated using Student t-test and are indicated as follow: *$P<0.05$, **$P<0.01$, ***$P<0.001$ (*i.e.* decrease relative to siCTRL). Cell lines are grouped according to their sensitivity to PRMT5 inhibitor EPZ015666.

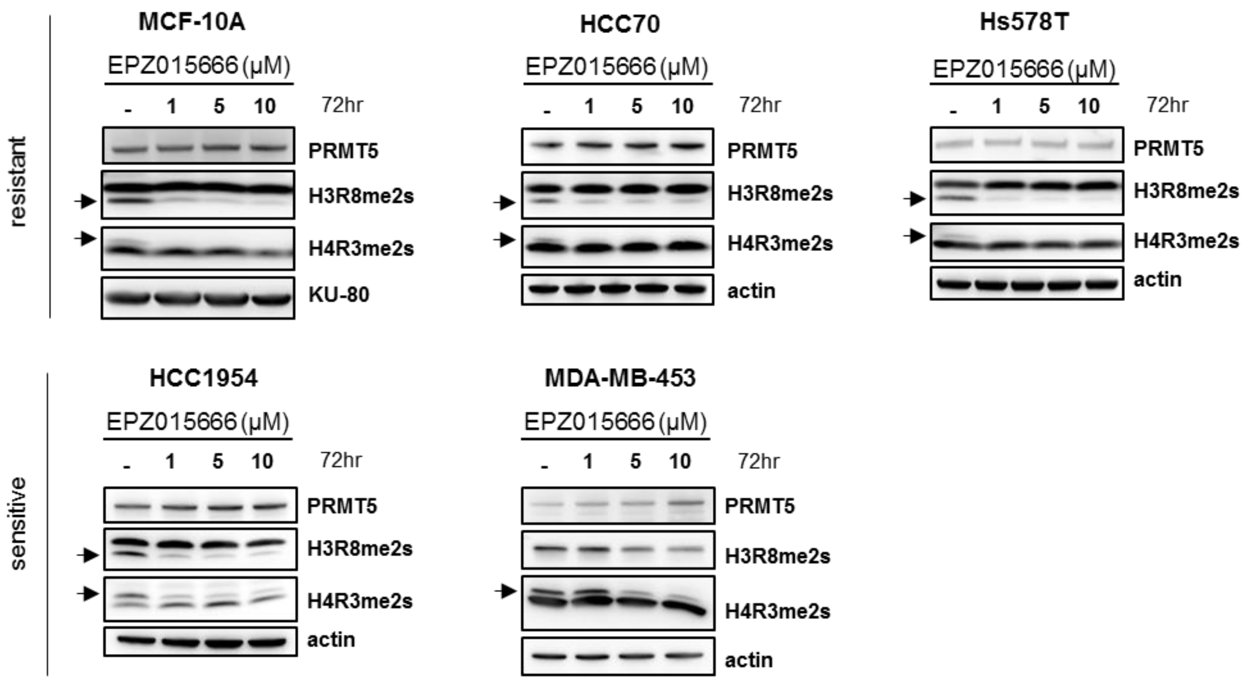

Supplementary Figure S5. EPZ015666 inhibits PRMT5 activity in breast cell lines.

Cells were treated with the indicated concentration of the PRMT5 inhibitor EPZ015666 or with vehicle (DMSO). PRMT5 activity was assessed 72 hours later by Western-Blot analysis using antibodies that recognize symmetric dimethyl-arginine on histones H3 (H3R8me2s) and H4 (H4R3me2s). PRMT5 expression was verified. Actin or KU-80 were used as loading controls. Pictures are from a single experiment representative of at least two independent experiments.

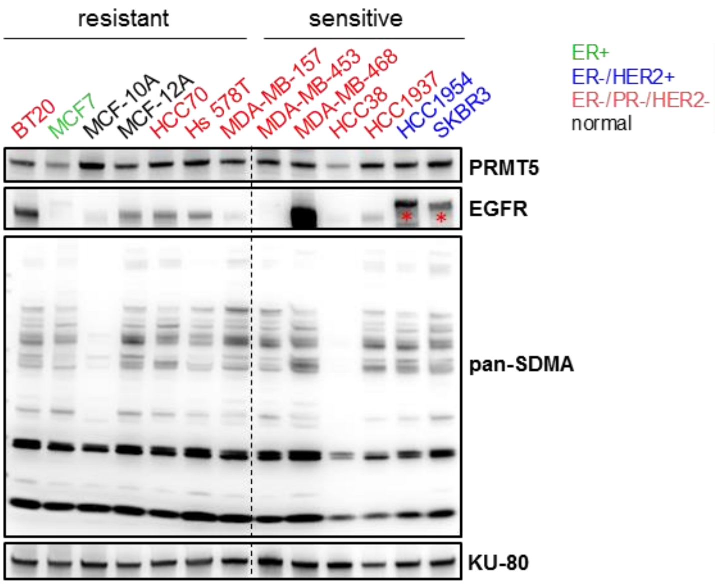

Supplementary Figure S6. PRMT5 expression, activity, and EGFR expression across a panel of breast cancer cell lines.

Western Blot analysis of PRMT5 expression and activity (total symmetric dimethylation of arginines; pan-SDMA), and of EGFR expression in a panel of breast cancer cell lines. KU-80 was used as a loading control. Cell lines are distributed according to their sensitivity to PRMT5 inhibitor EPZ015666. Breast cancer subtypes are indicated as follows: green (ER^+), blue (ER^-/$HER2^+$), red (ER^-/PR^-/$HER2^-$). The non-tumorigenic breast cells, MCF-10A and MCF-12A, are in black. Pictures are from a single experiment representative of three independent experiments. * corresponds to HER2, as the EGFR antibody used is known to cross-react with HER2.

a

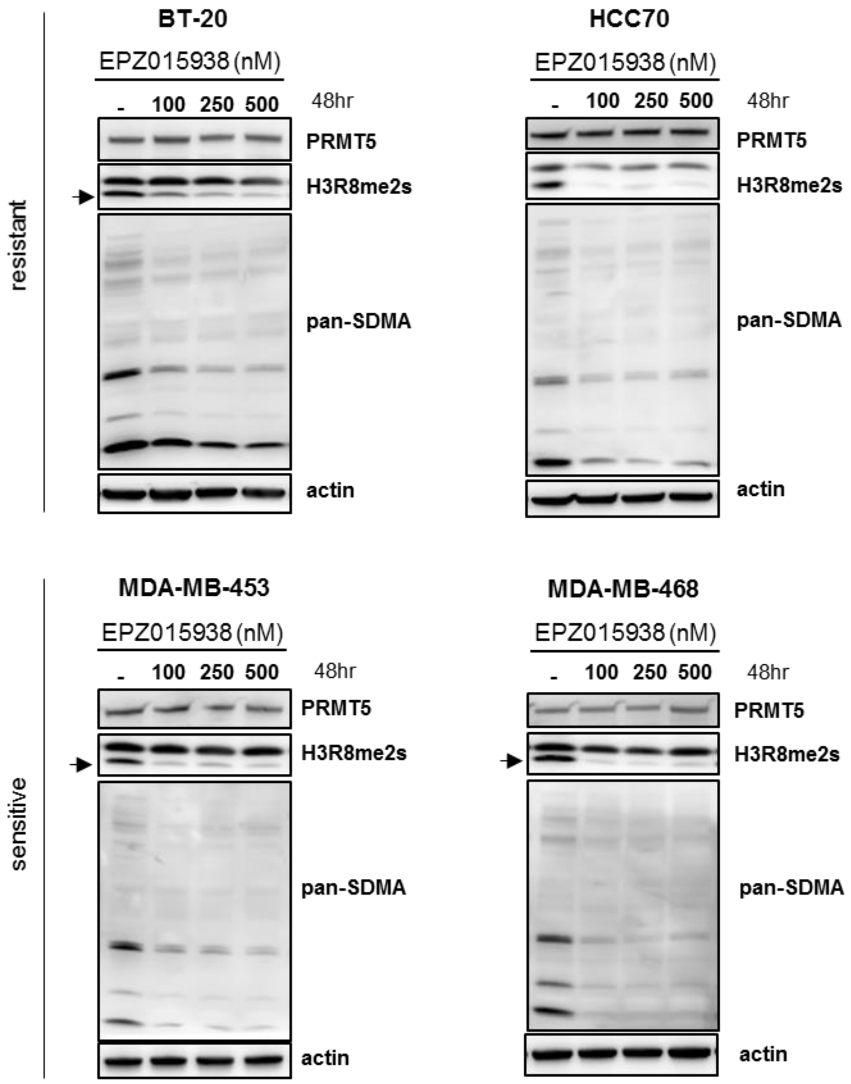
BT-20
HCC70
MDA-MB-453
MDA-MB-468
EPZ015938 (nM)
- 100 250 500
48hr
PRMT5
H3R8me2s
pan-SDMA
actin
resistant
sensitive

b

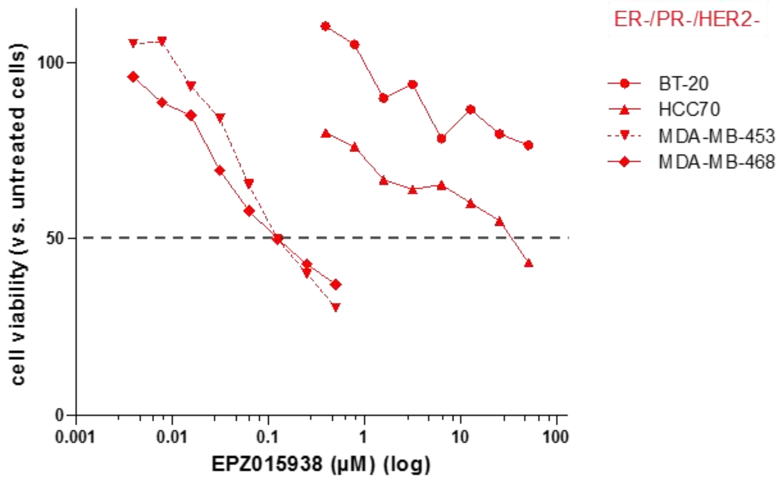
ER-/PR-/HER2-
BT-20
HCC70
MDA-MB-453
MDA-MB-468
cell viability (vs. untreated cells)
100
50
0
0.001
0.01
0.1
1
10
100
EPZ015938 (μM) (log)

Supplementary Figure S7. EPZ015038 inhibits PRMT5 activity of 4 TNBC cell lines and identifies sensitive and resistant cells lines

(*a*) EPZ015938 inhibits PRMT5 activity. Cells were treated with the indicated concentration of the PRMT5 inhibitor EPZ015938 or with vehicle (DMSO). PRMT5 activity was assessed 48 hours later by Western-Blot analysis using antibodies that recognize symmetric dimethyl-arginine on histones H3 (H3R8me2s) and total symmetric dimethyl-arginine (pan-SDMA). PRMT5 expression was verified. Actin was used as loading control. Pictures are from a single experiment representative of three independent experiments. (*b*) EPZ015938 identifies sensitive and resistant cells lines. Cell viability was determined by MTT assay after four doubling times. Results are expressed as the percentage of cell growth relative to untreated cells. The mean of at least three independent experiments for each cell line is represented.

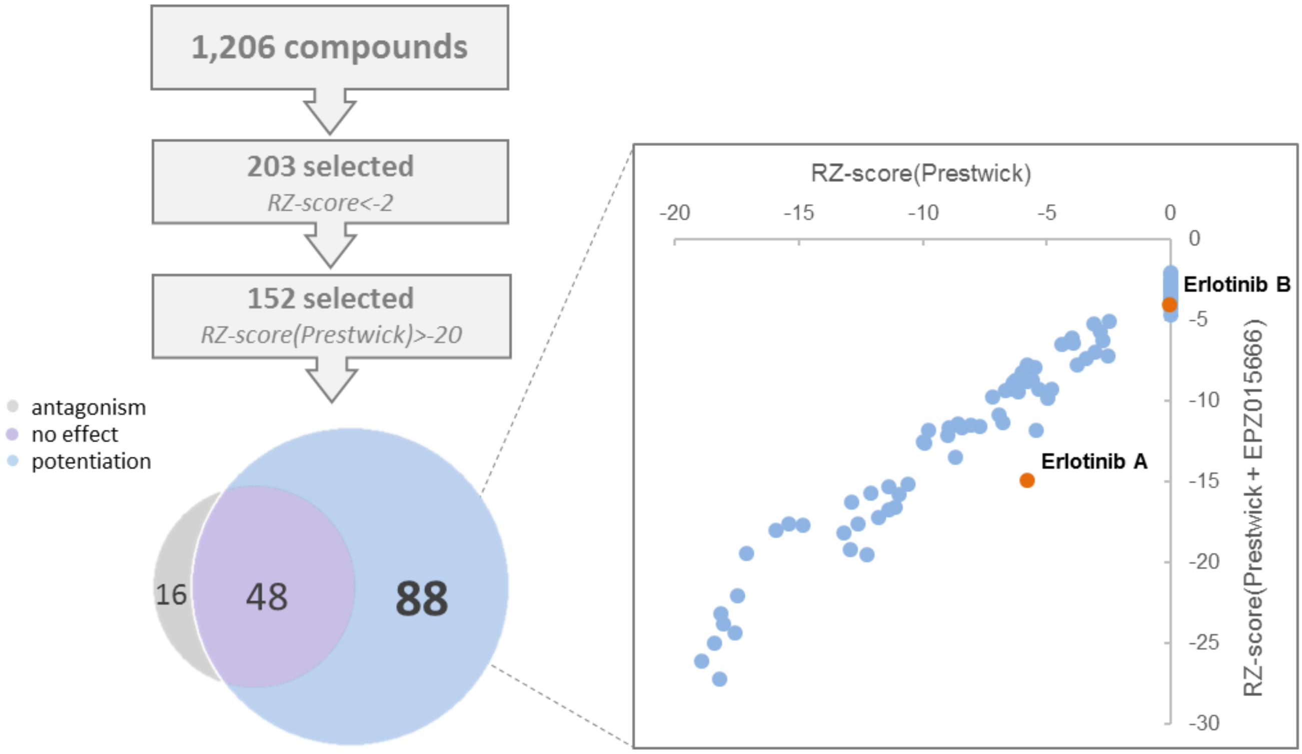

Supplementary Figure S8. Drug combination screen identifies Erlotinib as candidate for dual therapy with EPZ015666.

A screening with a panel of 1,200 FDA approved drugs (Prestwick Chemical Library) and six additional compounds including Erlotinib (Sup. Table 2) reveals a benefit to combining PRMT5 and EGFR inhibitors. MDA-MB-453 cells were seeded into 384-well plates and treated with the Prestwick Chemical Library (10µM) or one of six additional compounds (Sup. Table 2; concentration as indicated) alone, or in combination with EPZ015666 (10µM). After 3 days, cell viability was assessed by CellTiter-Glo (CTG) assay. Of the 1,206 screened compounds, 203 were identified as detrimental to MDA-MB-453 cell viability ($RZscore < -2$). Compounds which alone inhibited cell viability by 90% or more ($RZscore(Prestwick) \leq -20$) were excluded from this first selection, leaving 152 hits. The 152 hits were then sorted by $\Delta RZscore$ using the following thresholds to delineate the effect of the Prestwick+EPZ015666 combination: $\Delta RZscore > 0$: antagonism; $-2 < \Delta RZscore \leq 0$: no effect; $-2 \leq \Delta RZscore$: potentiation. Represented on the graphic are RZ-scores of Prestwick

alone vs. Prestwick+EPZ015666 for the 88 compounds for which the combination potentiates the effect of each compound alone, according to the described analysis. Erlotinib A (Prestwick Chemical) and Erlotinib B (Caiman Chemical) are highlighted in orange.

Supplementary Table 1. Antibodies used in this study.

Target	Cat. #	Manufacturer	Use
PRMT5	2252	CST	WB
PRMT5	ab109451	Abcam	IHC
c-casp7 (Asp198)	9491	CST	WB
c-casp8 (Asp391)	9496 (18C8)	CST	WB
c-PARP (Asp214) p89	ab32561	Abcam	WB
PARP/c-PARP (Asp214) p89	9546	CST	WB
pan-SDMA	13222	CST	WB
H3R8me2s	23613-0018	Epicypher	WB
H4R3me2s	ab5823	Abcam	WB
Actin Beta (Clone AC-15)	A5441	Sigma	WB
GAPDH	2118	CST	WB
KU-80 (C48E7)	2180	CST	WB
anti-Rabbit IgG (H+L) HRP	111-035-045	Interchim	WB
anti-Mouse IgG (H+L) HRP	115-035-062	Interchim	WB

Supplementary Table 2. Additional drugs to the Prestwick Chemical Library+EPZ015666 screening and concentration.

	**final concentration (µM)**
**Erlotinib (Caiman Chemicals)**	10
BI2536 **(PLK1 inhibitor - Selleck Chemicals)**	0.1
BAY1217389 **(TTK inhibitor - Selleck Chemicals)**	0.2
**Cisplatin (Mylan)**	10
**Doxorubicin (Sigma)**	0.1
**Paclitaxel (Selleck Chemicals)**	0.01

Supplementary Table 3. IC50 values for EPZ015666 in breast cancer cell lines. Cell lines are listed from most to least sensitive. IC50 values are calculated from at least three independent experiments. BC subtypes are indicated as follows: green (ER+), blue (ER-/HER2+), red (ER-/PR-/HER2-). The “normal” breast cells, MCF-10A and MCF-12A, are in black. ER-/PR-/HER2- cell lines encompass the different TNBC subtypes: BL1 (basal-like 1); BL2 (basal-like 2); LAR (luminal androgen receptor); MSL (mesenchymal stem like); UN (unclassified), as previously defined (2).

	ER-/PR-/HER2- subtype	**EPZ015666 IC50 (µM)**	*Standard deviation*	
HCC1954		**0.8**	*0.1*	**SENSITIVE**
MDA-MB-453	LAR	**1.0**	*0.3*	
HCC38	BL1	**2.2**	*1.6*	
MDA-MB-468	BL1	**2.2**	*0.9*	
MCF7		**2.6**	*1.0*	
SKBr3		**3.9**	*1.9*	
BT-20	UN	**12.3**	*7.8*	**RESISTANT**
HCC70	BL2	**29.9**	*5.7*	
MDA-MB-157	MSL	**33.4**	*1.7*	
MCF10A		**42.7**	*14.2*	
MCF12A		**47.1**	*11.2*	
Hs578T	MSL	**67.8**	*17.6*	
HCC1937	BL1	**74.7**	*32.4*	
